# CD4+CD25+Foxp3+ Regulatory T Cells Depletion May Attenuate the Development of Silica-Induced Lung Fibrosis in Mice

**DOI:** 10.1371/journal.pone.0015404

**Published:** 2010-11-03

**Authors:** Fangwei Liu, Jie Liu, Dong Weng, Ying Chen, Laiyu Song, Qincheng He, Jie Chen

**Affiliations:** Division of Pneumoconiosis, School of Public Health, China Medical University, Shenyang, People's Republic of China; New York University, United States of America

## Abstract

**Background:**

Silicosis is an occupational lung disease caused by inhalation of silica dust characterized by lung inflammation and fibrosis. Previous study showed that Th1 and Th2 cytokines are involved in silicosis, but Th1/Th2 polarization during the development of silicosis is still a matter of debate. Regulatory T cells (Treg cells) represent a crucial role in modulation of immune homeostasis by regulating Th1/Th2 polarization, but their possible implication in silicosis remains to be explored.

**Methodology/Principal Findings:**

To evaluate the implication of Treg cells in the development of silicosis, we generated the Treg-depleted mice model by administration of anti-CD25 mAbs and mice were exposed to silica by intratracheal instillation to establish experimental model of silica-induced lung fibrosis. The pathologic examinations show that the Treg-depleted mice are susceptive to severer inflammation in the early stage, with enhanced infiltration of inflammatory cells. Also, depletion of Treg cells causes a delay of the progress of silica-induced lung fibrosis in mice model. Further study of mRNA expression of cytokines reveals that depletion of Tregs leads to the increased production of Th1-cytokines and decreased production of Th2-cytokine. The Flow Cytometry and realtime PCR study show that Treg cells exert the modulation function both directly by expressing CTLA-4 at the inflammatory stage, and indirectly by secreting increasing amount of IL-10 and TGF-β during the fibrotic stage in silica-induced lung fibrosis.

**Conclusion/Significance:**

Our study suggests that depletion of Tregs may attenuate the progress of silica-induced lung fibrosis and enhance Th1 response and decelerate Th1/Th2 balance toward a Th2 phenotype in silica-induced lung fibrosis. The regulatory function of Treg cells may depend on direct mechanism and indirect mechanism during the inflammatory stage of silicosis.

## Introduction

Silicosis is characterized by lung inflammation and fibrosis [1], [2], which is caused by inhalation of silica particle, an oxide of silicon with a chemical formula of SiO_2_. Silicotic nodules are known as the major pathological character of silicosis. Silicosis has been studied extensively, yet little is known about the crucial cellular and molecular mechanisms that initiate and propagate the process of inflammation and scarring. Several evidences support that the pathogenesis of silicosis involves uncontrolled immune processes [3]. Alveolar macrophages, neutrophils, dendritic cells and many inflammatory mediators such as cytokines and chemokines are identified involved in the development of silicosis. Macrophages can be activated by silica particles, followed by the activation of the apoptosis associated speck-like protein- and NALP3-dependent inflammasome, which may trigger the production of IL-1β [4]–[6]. Upon silica particles stimulation, T lymphocytes were activated by antigen presenting cells (APC) such as dendritic cells (DC) and macrophages in the processing and presentation of silica antigen. Participation of CD4+ T cells, including Th1 and Th2 cells, in the pathogenesis of lung fibrosis induced by silica particles has been indicated in several studies [7]–[9]. Adaptive Th1/Th2 polarization during the development of silicosis is still a matter of debate. The role of Th1 immune response in silicosis has not been conclusively defined. Some studies showed that Th1 immune responses were dominant in the inflammatory stage of silicosis; however, contradictory findings on the functions of IFN-γ in silica-induced lung fibrosis have been reported [10]–[12]. There are several evidences supporting that silica-induced lung fibrosis is correlated with markers of a Th2-like response such as increased IL-4 levels [12]–[14]. IL-4-/- and IL-4Rα-/- mice did not, however, appear protected against the development of silicosis, suggesting that Th2 immune response is not essential for the development of this fibrotic disease [15]. Several evidences support that the pathogenesis of silicosis involves uncontrolled immune processes [3]. This may suggest that a more complex immunological mechanism, especially the modulation of immune homeostasis, exists in the development of silicosis. As a result, the cells, which modulated the immune homeostasis, appear to be significant for the development of silicosis.

Regulatory T cells (Treg cells) represent a crucial role in the maintenance of immune homeostasis in the airways. Naturally occurring CD4+CD25+ regulatory T cells regulate immune responses against external antigens via suppression of CD4+ and CD8+ T cells [16]–[19]. Major populations of regulatory T cells studied in the context of lung diseases are natural thymic-derived CD4+CD25+Foxp3+ Treg cells and peripherally antigen-induced CD4+ Treg cells, which comprise both Foxp3-positive and –negative populations [20], [21]. The mechanisms by which Treg cells inhibit the function of immune cells have been described [22]. A major inhibitory mechanism appears to be via anti-inflammatory cytokines such as IL-10 and TGF-β [23], [24], but inhibitory molecules such as CTLA-4 are also likely to contribute [22]. The suppression of Th1-mediated diseases by CD4+CD25+ Treg cells has been well documented [25], [26]. Human studies suggest that CD4+CD25+ Treg cells also suppress Th2-type diseases [27]–[29]; mouse studies have yielded contradictory results [30]–[34]. The percentage of peripheral blood CD4+CD25+ Foxp3+ Treg cells is slightly lower in silicosis patients (SILs) compared with healthy donors (HD) and the function of Treg cells in SILs is less significant than in HDs [35]. Treg cells are identified in the development of silicosis; however, the mechanism of CD4+CD25+Foxp3+ Treg cells to regulate immune homeostasis is still poorly understood in silicosis.

The objective of this study was 1) to investigate the role of Treg cells during inflammation and fibrosis in silicosis, 2) to study whether Treg cells regulate the process of silicosis by modulating the maintenance of immune homeostasis in the lung. We used anti-CD25 antibodies to neutralize regulatory T cells and assessed the immune responses of silica-induced lung fibrosis. We demonstrate that the depletion of Treg cells exacerbated the early lung inflammation and delayed the process of fibrosis and Th1/Th2 balance toward a Th2 dominant response in experimental model of silicosis.

## Results

### Enhancement of early lung inflammation in silica-induced lung fibrosis in mice by depletion of CD4+CD25+ regulatory T cells

**Figure 1:**
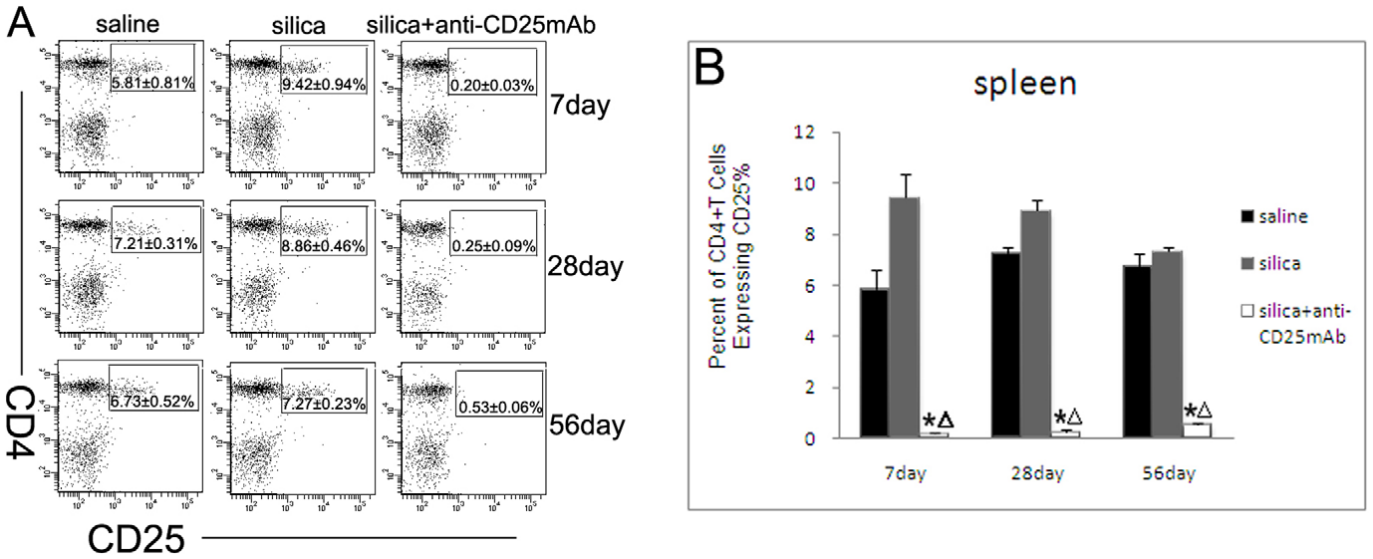
Injection of anti-CD25 mAb sufficiently depletes CD4^+^CD25^+^ regulatory T cells in vivo continuously. (A) C57BL/6 mice were treated i.p. with 100 µg anti-CD25 mAb or control IgG, the percentage of CD4^+^CD25^+^ Treg cells in the spleen was assayed by using anti-CD4 and CD25 mAb by flow cytometry. (n = 5) (B) Percentage of CD4^+^ T cells expressing CD25 was shown in the graph. (n = 5) (*, as compare with the saline control group, P<0.05; △, as compare with the silica group, P<0.05).

To test the potential suppression of silica-induced lung fibrosis in mice by endogenous CD4+CD25+ Treg cells, we generated CD25+ T cell-depleted C57BL/6 mice by injection of the anti-CD25 mAb PC61. We examined lung responses to silica in mice injected with saline, silica and silica + anti-CD25 mAb at day 7, 28 and 56. To check the efficiency of neutralizing anti-CD25 mAb, we assessed the percentage of CD4+CD25+ regulatory T cells in the spleen. According to Fig. 1A and B, injection of anti-CD25 mAb successfully depletes CD4+CD25+ regulatory T cells continuously even at day 56. The percentage of CD4+CD25+ fraction in silica-treated mice increased compared with saline-treated mice at day 7 (Fig. 1B). This fraction gradually reduced for chronic lung immune response induced by silica (Fig. 1).

**Figure 2:**
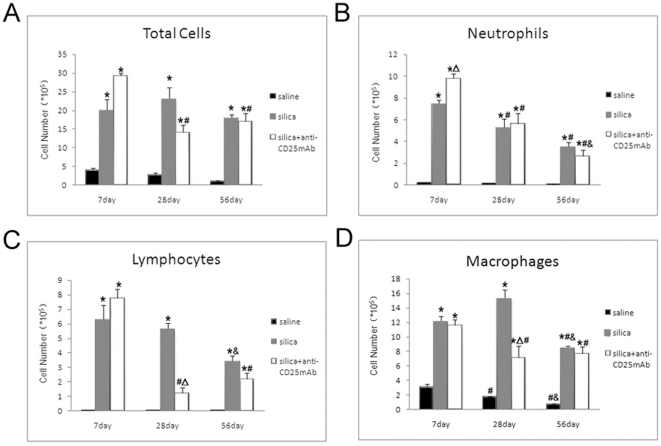
Depletion of Treg cells increases the accumulation of inflammation cells in lung response to silica. The total cells (A), neutrophils (B), macrophages (C) and lymphocytes (D) in BALF were counted by using Giemsa staining. (*, as compare with the saline control group, P<0.05; △, as compare with the silica group, P<0.05; #, as compare with 7day of the same group, P<0.05; &, as compare with 28day of the same group, P<0.05).

Early lung inflammation was significantly increased in silica-treated and Treg-depleted mice compared with saline-treated control animals, as measured at day 7, 28 and 56 by inflammatory cell counts in BALF (Fig. 2). We examined total cells, neutrophils, macrophages and lymphocytes in BALF. At day 7, more inflammatory cells were infiltrated in silica-treated and Treg-depleted mice (Fig. 2A). Treg cell ablation demonstrated significant reduction of total cells at day 28 and 56 compared with those at day 7 (Fig. 2A). Treg-depleted mice dramatically enhanced neutrophil accumulation (Fig. 2B). The neutrophil accumulation declined gradually in both silica-treated and Treg-depleted mice at day 28 and 56 (Fig. 2B). This suggested that Treg cells contributed to limit the severely acute neutrophil accumulations during the early lung inflammation. For chronic inflammatory responses, neutrophil accumulations were significantly limited in both silica- and Treg-treated mice at day 28 and 56 (Fig. 2B). The number of macrophages reduced clearly in Treg-depleted mice compared with silica-treated mice at day 28 (Fig. 2C). In silica-treated mice, the number of macrophages reached peak at day 28 and reduced at day 56 (Fig. 2C). At day 28, there was clearly reduced number of lymphocytes in Treg-depleted mice compared with silica-treated group (Fig. 2D).

### Depletion of CD4+CD25+ regulatory T cells decelerated the progress of silica-induced lung fibrosis in mice model

**Figure 3:**
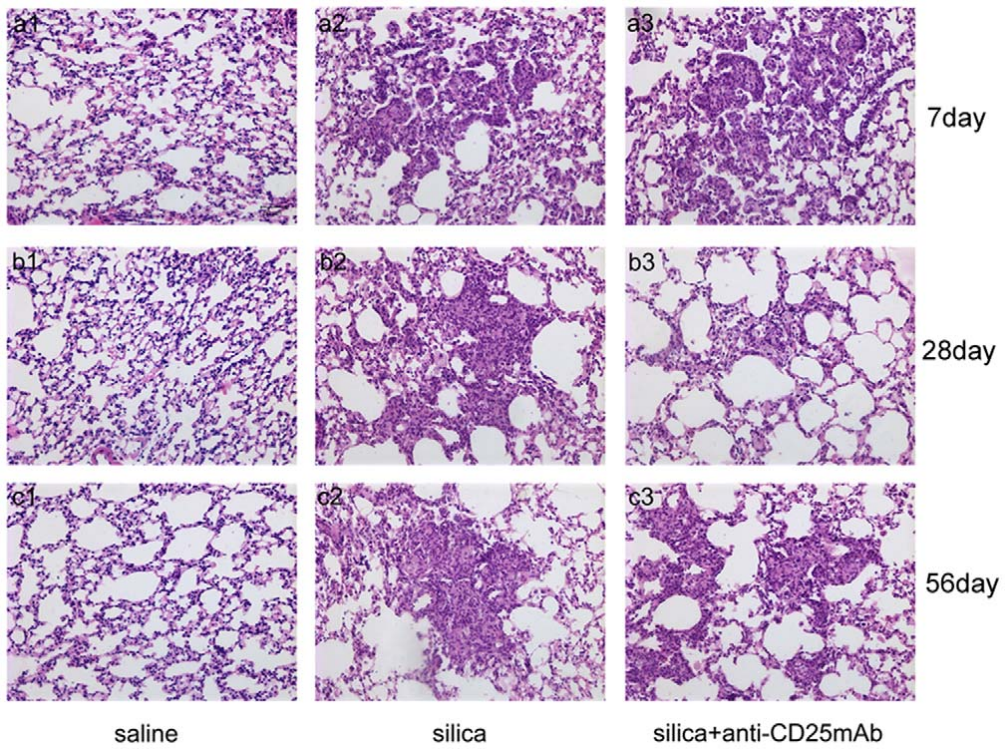
Histopathology changes in mouse lungs after instillation with HE staining (x200). a1-a3, day 7; b1-b3, day 28 and c1-c3, day 56. a1-c1, saline group; a2-c2, silica group; a3-c3,silica+anti-CD25mAb.

To investigate the role of CD4+CD25+ regulatory T cells in the pathogenesis of silica-induced lung fibrosis, the lung tissues of mice were observed by light microscope to monitor pathological changes and graded for silicotic nodule (Table 1). No obvious abnormalities were observed in the lungs of mice that received normal saline at day 7, 28 and 56 (Fig. 3a1, b1 and c1). In silica-treated group, there was a large infiltration of inflammatory cells and alveolar septal thickening in the lungs, and irregular cellular nodules were observed, at day 7 after silica instillation (Fig. 3a2). However, at day 7 in Treg-depleted group, significantly severer infiltration of inflammatory cells and alveolar septal thickening in the lungs, and more irregular cellular nodules were observed compared with that in silica-treated group (Fig.3a3). At day 28, the inflammation in the lungs of silica-treated mice was significantly extenuated. There were more and larger cellular nodules compared with day 7. Fibrotic cellular nodules were observed (Fig. 3b2). While in Treg-depleted group at day 28, inflammation was extenuated and there were a bit larger cellular nodules but reduced number of cells in the nodules (Fig. 3b3). At day 56, fibrotic cellular nodules increased and cellular fibrotic nodules appeared in silica-treated group (Fig. 3c2). In Treg-depleted group, occasionally a small amount of irregular cellular nodules and fibrotic cellular nodules at day 56 were observed (Fig. 3c3). According to the pathologic examination, depletion of Treg cells caused delay of the progress of silicosis but did not change the consequence (Table 1 and Fig. 3).

**Table 1:** Silicotic nodule of the mice lungs in each group at day 7, 28 and 56.

Groups	7 day after instillation		28 day after instillation		56 day after instillation	
	n	silicotic nodule grade	n	silicotic nodule grade	n	silicotic nodule grade
saline control	5	0	5	0	5	0
silica	5	I	5	I+∼II	5	II+∼III
Silica+anti-CD25mAb	5	I	5	I∼I+	5	I∼II

### Depletion of CD4+CD25+Foxp3+ regulatory T cells enhanced Th1 response and decelerated Th1/Th2 balance toward a Th2 phenotype in mice model of silica-induced lung fibrosis

**Figure 4:**
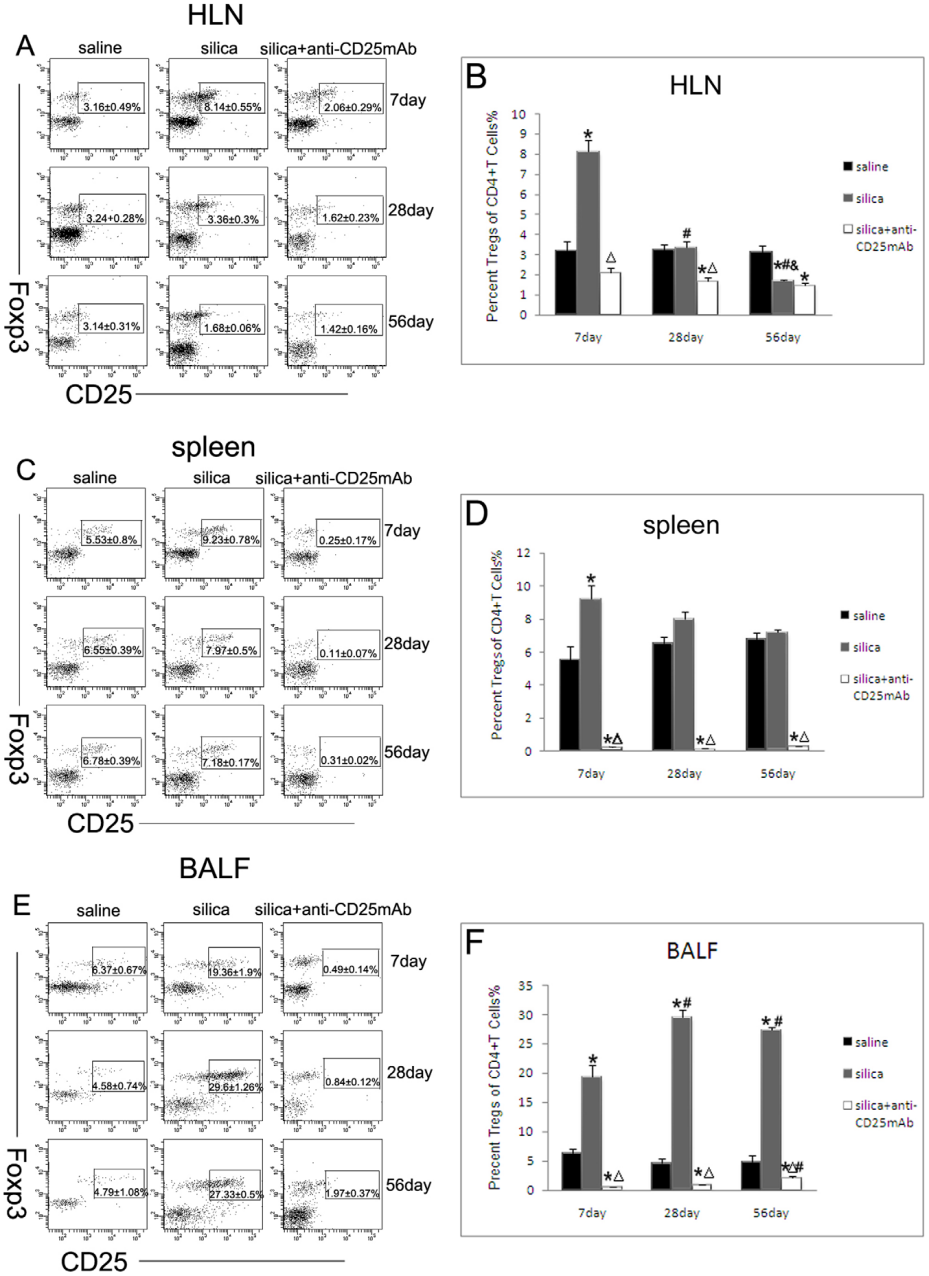
CD4+CD25+FOXP3+ T cells were the functional fraction of Treg cells in the silica-induced lung fibrosis. Treg cells in HLN (A), spleen (C) and BALF (E) were calculated by flow cytometry. (n = 5) The percentage of CD4+ T cells expressing CD25 and FOXP3 was shown in the graph (B, HLN; D, spleen; F, BALF) (n = 5) (*, as compare with the saline control group, P<0.05; △, as compare with the silica group, P<0.05; #, as compare with 7day of the same group, P<0.05; &, as compare with 28day of the same group, P<0.05).

Foxp3 expressed in peripheral CD4+CD25+ T cells and played an important role in the development and function of CD4+CD25+ Treg cells. To investigate the role of CD4+CD25+Foxp3+ regulatory T cells in silica-induced lung fibrosis, we first assessed the population of CD4+CD25+Foxp3+ T cells in HLN, spleen and BALF. The injection of anti-CD25 Abs neutralized most of the CD4+CD25+Foxp3+ fraction and these were consistent with the depletion of CD4+CD25+ T cells (Fig. 4). At day 7, the population of CD4+CD25+Foxp3+ T cells in HLN increased significantly in silica-treated mice compared with saline-treated control mice (Fig. 4A and B). In spleen, CD4+CD25+Foxp3+ T cells increased significantly in silica-treated mice compared with saline-treated control group at day 7 (Fig. 4C and D). This fraction reduced gradually at day 28 and 56 in silica-treated group (Fig. 4C and D). The percentage of CD4+CD25+Foxp3+ T cells in silica-treated mice also increased clearly in BALF compared with saline control mice at day 7, 28 and 56 (Fig. 4E and F). CD4+CD25+Foxp3+ T cells reached peak at day 28 and reduced at day 56 in silica-treated group, but were still significantly higher than those at day 7 (Fig. 4E and F).

The confocal immunofluorescence assay showed that the CD4+Foxp3+ T cells were identified in the lung tissue sections. The CD4+Foxp3+ T cells were observed in and around the cellular nodules, and the amount of the CD4+Foxp3+ T cells in silica-treated group is slightly more than that in Treg-depleted group at day 7 (Fig. S1a2 and Fig. S1a3). At day 28 and 56, the amount of the CD4+Foxp3+ T cells in both silica-treated and Treg-depleted group reduced obviously, and the most of the CD4+Foxp3+ T cells were around the nodules and little was in the nodules (Fig. S1).

**Figure 5:**
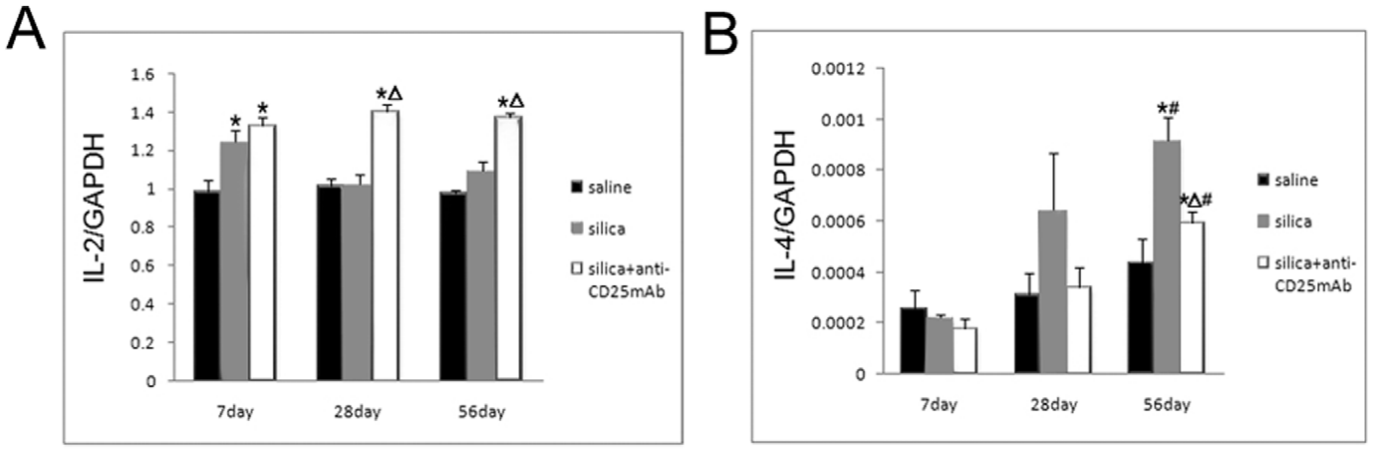
Treg cells affect the Th1/Th2 balance in the mice model of silica-induced lung fibrosis. Typical Th1 (IL-2) (A) and Th2 (IL-4)(B) cytokines were assayed by realtime RT-PCR by using -△△Ct method. (n = 5) (*, as compare with the saline control group, P<0.05; △, as compare with the silica group, P<0.05; #, as compare with 7day of the same group, P<0.05).

Next we want to investigate the regulatory role of Treg cells in silica-induced lung fibrosis in mice. We extracted total RNA from lung homogenates and applied samples for realtime RT-PCR. Typical Th1 (IL-2, IFN-γ) and Th2 (IL-4) cytokines were studied. At day 7, both silica-treated and Treg-depleted group showed higher level of IL-2 expression compared with saline control group (Fig. 5A). The level of IL-2 in silica-treated group reduced to the same level with saline control group at day 28 and 56 (Fig. 5A). In Treg-depleted group, the level of IL-2 was still significantly higher than those in the other two groups at day 28 and 56 (Fig. 5A). In silica-treated group, the level of IL-2 increased clearly at the early inflammatory stage of silicosis (day 7) and decreased during the fibrotic reaction of silicosis (day 28 and 56). The level of IFN-γ in Treg-depleted group was significantly higher than that of silica-treated group at day 7, 28 and 56, and in both Treg-depleted and silica-treated group, the expression of IFN-γ was more than the saline group (Fig. S2). Depletion of CD4+CD25+Foxp3+ regulatory T cells enhanced Th1 response at the later stage of lung fibrosis. These data suggested that CD4+CD25+Foxp3+ regulatory T cells could suppress Th1 response during the early inflammation period of silica-induced lung injury.

The level of IL-4 increased gradually during the process of silicosis and reached peak at day 56 in silica-treated group (Fig. 5B). These data were consistent with previous. At day 56, the level of IL-4 in Treg-depleted group was higher than that in saline control group but lower than that in silica-treated group (Fig. 5B). Depletion of CD4+CD25+Foxp3+ regulatory T cells limited and delayed the Th2 responses in silica-induced lung fibrosis. These suggested that CD4+CD25+Foxp3+ regulatory T cells played a regulatory role in silica-induced lung fibrosis by modulating Th1/Th2 balance.

### The regulatory function of CD4+CD25+ Foxp3+ regulatory T cells in silica- induced lung fibrosis in mice depends on both direct and indirect mechanism

**Figure 6:**
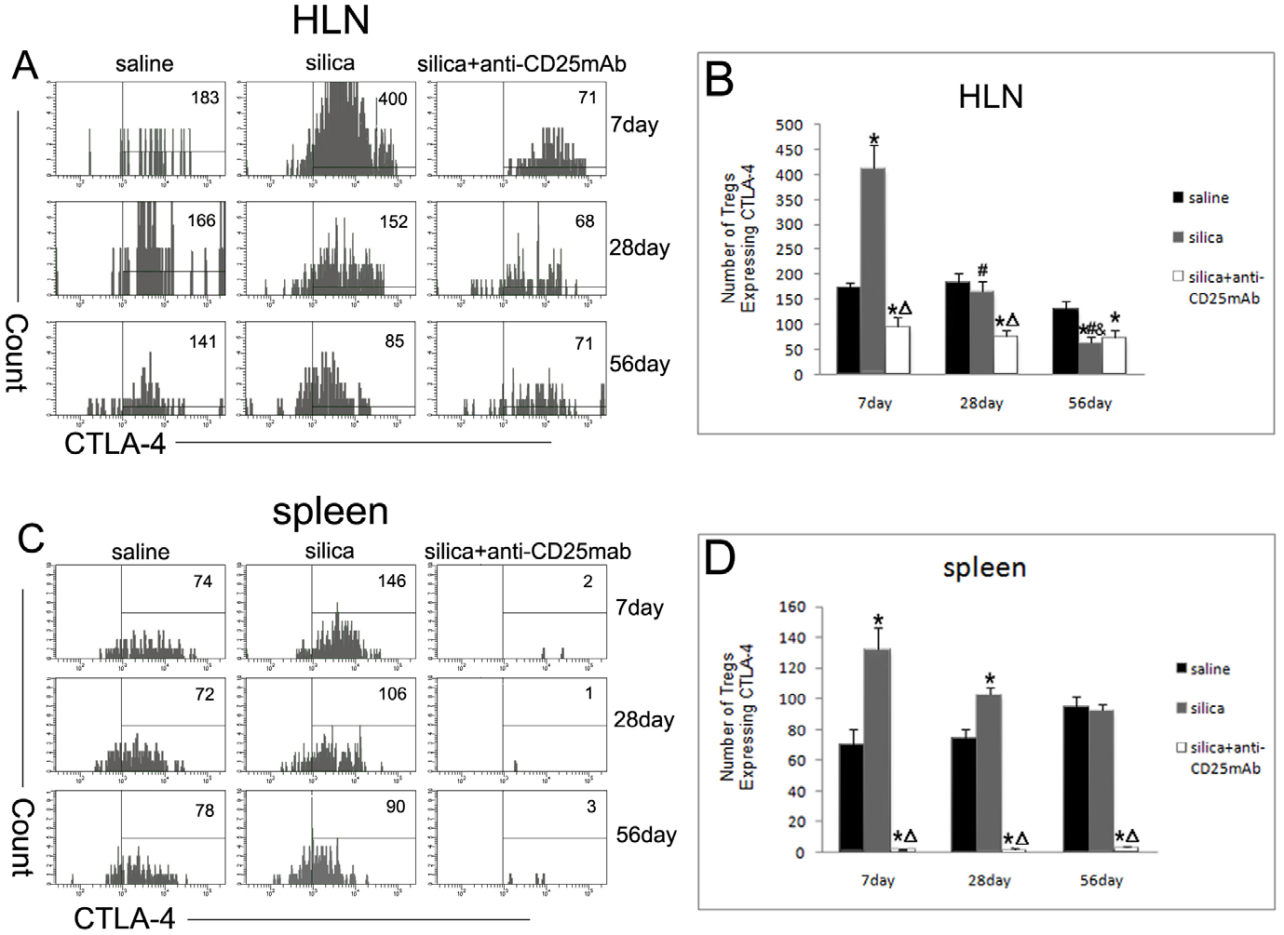
The expression of CTLA-4 in Treg cells in the HLN and spleen. CTLA4 expressed in CD4+CD25+FOXP3+ Treg cells of HLN (A) and spleen (C) was assayed by flow cytometry. (n = 5) The number of Treg cells expressing CTLA4 of HLN (B) and spleen (D) was shown in the graph. (n = 5) (*, as compare with the saline control group, P<0.05; △, as compare with the silica group, P<0.05; #, as compare with 7day of the same group, P<0.05; &, as compare with 28day of the same group, P<0.05).

We next sought about the mechanisms of CD4+CD25+Foxp3+ regulatory T cells in silica-induced lung fibrosis. First, we checked the expression of CTLA-4 in HLN and spleen. The expression of CTLA-4 in Treg-depleted mice reduced clearly and was consistent with the depletion of CD4+CD25+ T cells (Fig. 6). In silica-treated mice, the number of Treg cells expressing CTLA-4 increased significantly at day 7 compared with saline control mice in both HLN and spleen (Fig. 6). In HLN, the numbers of Treg cells expressing CTLA-4 in silica-treated mice declined to the similar level of those in saline control mice at day 28 (Fig. 6A and B). At day 56, the numbers of Treg cells expressing CTLA-4 in silica-treated mice were less than those in saline control mice in HLN (Fig. 6A and B). The silica-treated mice still showed more Treg cells expressing CTLA-4 compared with saline control mice in spleen at day 28 while no difference at day 56 (Fig. 6C and D).

**Figure 7:**
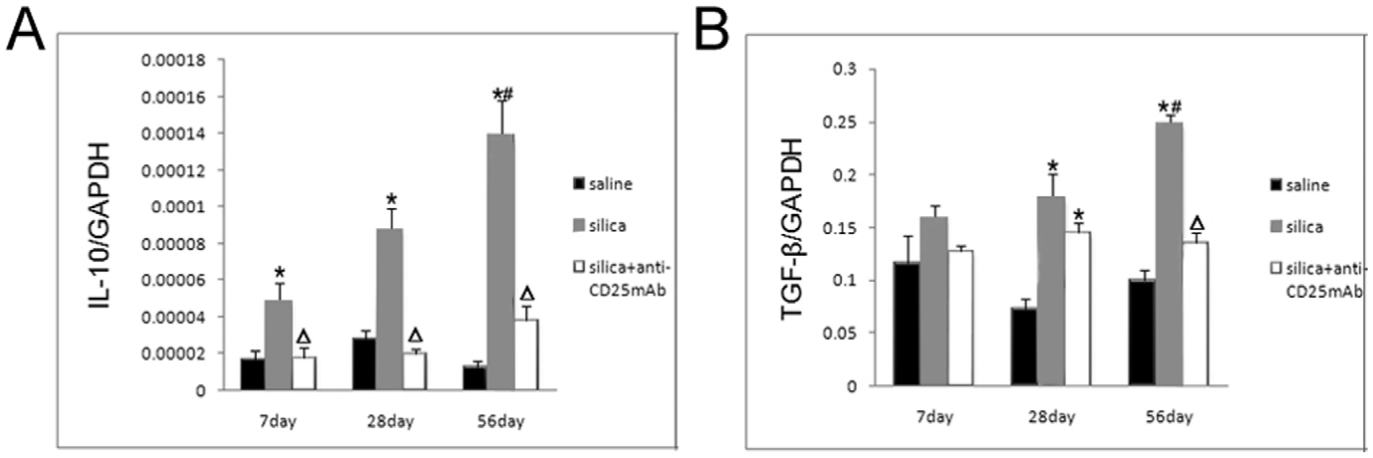
The function of Treg cells depends on both IL-10 (A) and TGF-β (B). The IL-10 and TGF-β mRNA were assayed by realtime RT-PCR by using -△△Ct method. (n = 5) (*, as compare with the saline control group, P<0.05; △, as compare with the silica group, P<0.05; #, as compare with 7day of the same group, P<0.05; &, as compare with 28day of the same group, P<0.05).

To investigate the regulatory role of Treg cells in silica-induced lung fibrosis of mice mould, we next explored whether Treg cells regulated the immune homeostasis through IL-10 and/or TGF-β during the short- and long-term lung responses to silica particles. At day 7, 28 and 56, we found a significantly increased lung expression of IL-10 and TGF-β mRNA in lung homogenates from silica-treated mice compared with saline controls (Fig. 7A and B). Depletion of Treg cells caused significantly reduction of IL-10 expression compared with silica-treated mice (Fig. 7A). The expression of TGF-β in Treg-depleted mice kept in a similar level at day 7, 28 and 56 (Fig. 7B). At day 28, expression of TGF-β mRNA in silica-treated and Treg-depleted mice was significantly higher than that in saline controls (Fig. 7B).

## Discussion

Silicosis, an occupational lung disease, is caused by inhalation of crystalline silica dust. The pathogenesis of silicosis involves alveolar cell injury and activation followed by cytokine signaling and cell recruitment in the areas of silica dust deposition [1], [2]. In this study, we demonstrated that Treg cells regulated immune responses against silica particles via suppression of inflammatory cells in the early stage. Treg cells may modulate Th1/Th2 balance toward a Th2 dominant response by suppressing Th1 response in experimental model of silica-induced lung fibrosis. Depletion of Treg cells decelerated the progress of abnormal repair and fibrosis against silica particles.

In this study, first we generated CD25+ T cell-depleted C57BL/6 mice by injection of the anti-CD25 mAb PC61 and examined lung responses to silica in mice. CD4+CD25+Treg cells were depleted continuously by injection of anti-CD25 mAb (PC61). Since the recently activated effector T cells may also express CD25 transiently, it is difficult to exclude the possibility that the mAb related to depletion of activated T cells. Despite that the anti-CD25 mAb used in our study has also been used in other studies to transiently deplete Treg cells [36], [37]. There is also a study showed that the anti-CD25 mAb selectively depleted cells with highest level of CD25 expression, and the changed intensity of immune response was more consistent with the depletion of Treg cells than that of effector T cells [37]. In our study, the percentage of CD4+CD25+ Treg cells increased at early stage and gradually reduced later in silica-treated group. This suggested that naturally occurred Treg cells played some roles in silica-induced lung fibrosis. We then examined lung responses to silica by counting the inflammatory cells in BALF of mice injected with saline, silica and silica + anti-CD25 mAb at day 7, 28 and 56. Depletion of CD4+CD25+ Treg cells demonstrated drastic enhancement of early lung inflammation, especially accumulation of neutrophils. Lung neutrophils have an important role in the early stage of inflammation in response to silica particles. Our results suggested that Treg cells contributed to limit the severely acute accumulation of inflammatory cells especially neutrophils in the early stage to silica.

Next we examined the pathology of lung tissues. Silicotic nodules were graded as follows: cellular nodules as Stage I; fibrotic cellular nodules as Stage II; cellular fibrotic nodules as Stage III; fibrotic nodules as Stage IV. At the early stage, the infiltration of inflammatory cells was observed in silica group. However, significantly severer infiltration of inflammatory cells, alveolar septal thickening and more irregular cellular nodules were observed in Treg-depleted group. The severer infiltration of inflammatory cells was consistent with the enhancement of inflammatory cell by counting. Later after challenged by silica, the silicotic nodule grade was a little higher in silica group than in Treg-depleted group. In silica group, fibrotic cellular nodules increased and cellular fibrotic nodules even appeared. However, only a small amount of irregular cellular nodules and fibrotic cellular nodules could be observed occasionally in Treg-depleted group. Depletion of Treg cells actually delayed the progress of lung fibrosis, but did not prevent the consequence of silica-induced lung fibrosis. This result provided a new insight to slow down the development of silicosis by using anti-CD25 antibody.

To determine the mechanism of Treg cells in silica-induced lung fibrosis, we first examined the effective fraction (CD4+CD25+Foxp3+ Treg cells) in HLN, spleen and BALF. Most of the CD4+CD25+Foxp3+ fraction were neutralized and these results were consistent with that the anti-CD25 mAb selectively depleted those cells expressing the highest levels of CD25, a population recently demonstrated to comprise primarily Foxp3+ cells [38], [39]. At early stage (day 7), the CD4+CD25+Foxp3+ T cells in HLN and spleen of silica group increased significantly. This fraction in silica group reduced gradually at the later stage (day 28 and 56). The location of the CD4+Foxp3+ Treg cells in lung tissue slices was in and around the cellular nodules at early inflammatory stage, but around the cellular nodules at the late fibrosis stage. And the amount of the CD4+Foxp3+ Treg cells reduced at the late stage, which was consistent with the results of the depletion Treg cells. These results suggested that CD4+CD25+Foxp3+ Treg cells were the mainly effective fraction in response to silica particles.

Secondly, we want to investigate the regulatory role of CD4+CD25+Foxp3+ Treg cells in silica-induced lung fibrosis by studying the mRNA expression of typical Th1 (IL-2, IFN-γ) and Th2 (IL-4) cytokines. Both silica group and Treg-depleted group secreted high level of IL-2 at early stage. The level of IL-2 in silica group reduced to the same level with saline control group at late stage, however, in Treg-depleted group, the level of both IL-2 and IFN-γ was still significantly higher than that of silica group, which suggested depletion of CD4+CD25+Foxp3+ regulatory T cells caused failure to modulate the Th1/Th2 balance via suppression of Th1 response at the later stage of lung fibrosis. Besides, in silica-treated mice, the level of IL-4 increased gradually during the process of silicosis and reached peak at day 56, which suggested that Th2 immune response played some role during the fibrotic process of silicosis. However, the expression of IL-4 in Treg-depleted group was not increased as obviously as that in silica group. Some researchers thought that Th2 immune response was not essential for the development of this fibrotic disease [15]. And in our study, depletion of CD4+CD25+Foxp3+ regulatory T cells indeed limited and delayed the Th2 responses in silica-induced lung fibrosis. In all, our data suggested that CD4+CD25+Foxp3+ regulatory T cells could modulate Th1/Th2 polarization by suppressing Th1 response during the early lung inflammation, and played a regulatory role by modulating Th1/Th2 balance toward a Th2 dominant response.

The mechanisms by which Treg cells inhibit the function of effector T cells and other immune cells have been described [22]. A major inhibitory mechanism appears to be via anti-inflammatory cytokines such as IL-10 and TGF-β [23], [24], but inhibitory molecules such as CTLA-4 are also likely to contribute [22]. The suppression of Th1-mediated diseases by CD4+CD25+ Treg cells has been well documented [25], [26]. Human studies suggest that CD4+CD25+ Treg cells also suppress Th2-type diseases [27]–[29]; mouse studies have yielded contradictory results [30]–[34]. In our study, the CTLA-4 expression of CD4+CD25+Foxp3+ Treg cells in Treg-depleted mice reduced clearly and was consistent with the depletion of CD4+CD25+ T cells. In silica-treated mice, the number of Treg cells expressing CTLA-4 increased significantly at the early phase and decreased gradually late in both HLN and spleen. In HLN, the numbers of Treg cells expressing CTLA-4 in silica-treated mice were even less than those in saline control mice at day 56. These suggested that during the inflammatory stage of silicosis, Treg cells suppressed the inflammation and Th1 immune response directly by expressing CTLA-4 molecule.

Next we explored whether Treg cells regulated the immune homeostasis through IL-10 and/or TGF-β during the short- and long-term lung responses to silica particles. A significantly increased lung expression of IL-10 and TGF-β mRNA in lung homogenates from silica-treated mice was observed through the whole process of silica-induced lung fibrosis. And depletion of Treg cells caused significantly reduction of IL-10 expression, and the expression of TGF-β kept in a similar level. These results suggested that Treg cells also modulated the immune response against silica indirectly by secreting IL-10 and TGF-β. At the early stage of silicosis, Treg cells mainly inhibited the inflammation by CTLA-4 molecule. With the development of silicosis, Treg cells suppress Th1 immune response by secreting increasing amount of IL-10 and TGF-β. Then Th1/Th2 polarization shift toward a Th2 dominant immune response.

Altogether, our study showed that depletion of CD4+CD25+ regulatory T cells decelerated the progress of silica-induced lung fibrosis and this might provide a new insight in limit the process of fibrosis for silicosis patients by using anti-CD25 Abs. Depletion of CD4+CD25+Foxp3+ Treg cells enhanced Th1 response and decelerated Th1/Th2 balance toward a Th2 phenotype in silica-induced lung fibrosis. These suggested that Treg cells regulated the inflammation against silica via suppression of inflammatory cells in the early stage and Treg cells may modulate Th1/Th2 polarization toward a Th2 dominant response by suppressing Th1 response in experimental model of silica-induced lung fibrosis. The regulatory function of CD4+CD25+Foxp3+ Treg cells in silica-induced lung fibrosis depends on direct mechanism at the inflammatory stage and indirect mechanism during the fibrotic stage in silica-induced lung fibrosis.

## Materials and Methods

### Animal

Female C57BL/6 mice were purchased from SLAC Laboratory animal co.LTD. (Shanghai, CHINA) at 6–8 weeks of age. All animals were housed in a specific pathogen-free environment and maintained on standard mouse chow at an environmental temperature of 24±1°C and a 12/12 h light/dark cycles and water ad libitum. All animal experiments were approved by the Animal Care and Use Committee at the China Medical University with a permit number of CMU62043003, which complies with the National Institute of Health Guide for the Care and Use of Laboratory Animals.

### Silica preparation

Silica was purchased from Sigma (St., Louse, MO, USA). The content of the freeing SiO_2_ dust was >99%, and the particle size of 80% SiO_2_ dust was between 1 to 5 µm. Silica was grinded in saline for 3 hours, boiled in 1N HCl, washed, dried, suspended in sterile saline. Suspensions were sonicated for 10 min before use.

### Silica exposure

45 mice were randomly divided into three groups (n = 15) as follows: the silica+anti-CD25mAb group, silica group and the saline control group. Animals were anesthetized with intraperitoneal injection of 2% pentobarbital sodium 45 mg/kg body weight. The trachea was exposed by opening the neck skin and blunt dissection. A 7-gauge needle was used to insert into the trachea trans-orally. Mice received either the suspension of 3 mg silica in a total volume of 100 µl sterile saline or just the sterile physiological saline in same volume. The site of surgery was sutured and cleaned with penicillin and the mice were allowed to recover until they were sacrificed.

### CD4+CD25+ regulatory T cell depletion

Mice from silica+anti-CD25mAb group or silica group and saline control group received intraperitoneal injection with 100 µg of anti-CD25 mAb (PC61) (BioLegend, 11080 Roselle Street, San Diego, CA 92121) or rat IgG1 (BioLegend, 11080 Roselle Street, San Diego, CA 92121) in phosphate buffered saline just one day before the silica exposure. And repeatedly treated i.p. with PC61 100 µg or rat IgG1 of the same volume every 7 days after the silica exposure for continuing depletion.

### Bronchoalveolar lavage and differential cell counts

Mice were sacrificed at 7, 28 or 56 days after challenged by silica instillation or sterile physiological saline. The lungs were removed and washed in cold PBS. Bronchoalveolar lavage fluid (BALF) was obtained by cannulating the trachea, injecting and retrieving 1 ml aliquots of sterile physiological saline for 3 times. The BALF was centrifuged at 1500 rpm for 8 min at 4°C. After lysis of RBC, the BAL cell pellet was washed and resuspended in PBS. The total cell counts were determined using standard hematologic procedures. Cytospin of BAL was prepared and stained with the Wright-Giemsa method. Macrophage, neutrophil or lymphocytes were identified on 200 cells using standard morphologic criteria.

### Pathological examination

Lung was fixed in 4% paraformaldehyde -PBS. The tissue was embedded in paraffin, cut in 6 µm-thick sections. The tissue sections were stained with hematoxylin and eosin (H&E). Silicotic nodules were graded as follows: cellular nodules as Stage I; fibrotic cellular nodules as Stage II; cellular fibrotic nodules as Stage III; fibrotic nodules as Stage IV.

### Confocal immunofluorescence assay

Following extensively rinsing with 0.01 M PBS (pH 7.4) for two times, the slides of lung tissue were blocked with goat serum (Histostain-Plus Kits, ZSBG-BIO) for 30 min to reduce nonspecific binding, then incubated with a polyclonal RBITC-conjugated rabbit anti-CD4 antibody 40 µg/ml (Beijing biosynthesis biothechnology) for 2 h at room temperature. After washing with PBS for three times, the slides were incubated with a polyclonal FITC conjugated rabbit anti-Foxp3 antibody 10 µg/ml (Beijing biosynthesis biothechnology) for 2 h at room temperature. The localization of CD4+Foxp3+T cells was captured by a confocal laser scanning microscope (TCS sp2/AOBS,LEICA) and analyzed with the leica confocal software package.

### Purification of hilar lymph nodes and spleen cells

The hilar lymph nodes (HLN) were harvested and dissected by using needles then digested with 0.25% trypsin for 5 min at 37°C. 3% fetal bovine serum-PBS was used to end the digestion. Centrifuge it at 1500 rpm for 8 min at 4°C. The HLN cell pellet was washed and resuspended in PBS. The spleens were removed, grinded and mechanically dissociated in cold PBS. After lysis of RBC, spleen cells were washed and resuspended in PBS.

### Flow cytometry

Analysis of cell surface marker expression was performed using a FACSCantoII (BD, Franklin Lakes, NJ USA) system. Briefly, the cells from BALF, HLN and spleen were resuspended in PBS and blocked with purified rat anti-mouse CD16/CD32 (BD Pharmingen, San Jose, CA, USA) for 10 min at 4°C. Cells were then incubated with anti-mouse PerCP-conjugated CD3 (BD Pharmingen, San Jose, CA, USA), anti-mouse PE-Cy7-conjugated CD4 (BD Pharmingen, San Jose, CA, USA), and anti-mouse APC-conjugated CD25 (BD Pharmingen, San Jose, CA, USA) for 20 min at 4°C in the dark. After cellular surface staining, cells were washed twice with 3% FBS-PBS. 1 ml working solution (0.25 ml fixation/permeabilization concentrate and 0.75 ml fixation/permeabilization diluent) (eBioscience, San Diego, CA 92121,USA))was added to fix and permeate the cell membrane for 30 min at 4°C in the dark. To label the nuclear factor Foxp3, cells were incubated with anti-mouse FITC-conjugated Foxp3 (eBioscience, San Diego, CA 92121, USA) for 1 h at 4°C in the dark. In addition, cells from NLH and spleen were incubated with anti-mouse PE -conjugated CTLA-4 (BD Pharmingen, San Jose, CA, USA) for 1 h at 4°C in the dark. After then, cells were washed twice with 3% FBS-PBS and re-suspended in 1% paraformaldehyde-PBS. Dead cells were gated out depending on forward scattering (FSC) and side scattering (SSC). Cells were analyzed with Diva software.

### RNA extraction and realtime RT-PCR

Total RNA was isolated from lung homogenates using the TRIZOL® Reagent (Invitrogen, Carlsbad, CA, USA) according to the manufacturer's protocol. The RNA concentration and the ratio of A 260/280 of were determined by UV spectrophotometer.

The primers and the Taqman probes were designed with the Primer3 (http://frodo.wi.mit.edu/primer3) and the sequences were blasted (http://www.ncbi.nlm.gov/BLAST/). PrimeScript RT- PCR kit (DRR061A, Takara, Japan) was used for realtime RT- PCR. primer sequences were as follows: IL-2, sense 5′-TTGAGTGCCAATTCGATGATGAG-3′, antisense 5′-TTGAGATGATGCTTTGACAGAAGG-3′; IFN-γ, sense 5′- AAGCGTCATTG AATCACACCTG -3′, antisense 5′- TGACCTCAAACTTGGCAATACTC -3′; IL-4, sense 5′-AAAATCACTTGAG AGAGATCATCGG-3′, antisense 5′-GTTGCTGTGAGGACGTTTG G-3′;IL-10, sense 5′-GGGGCCAGTACAGCCGGGAA-3′, anti-sense 5′-CTGGCTGAAGG CAGTCCGCA-3′;TGF-β, sense5′-TGTGGAACTCTACCAGAAATATAGC-3′, anti-sense 5′-GAAAGCCCTGTATTCCGTCTC-3′; GAPDH, sense5′-CAATGTGTCCGTCGTGGAT CT-3′, anti-sense 5′-GTCCTCAGTGTAGCCCAAGATG-3′. The probe sequences were as follows: IL-2, 5′-(FAM) CCTCAGAAAGTCCACCACAGTTGCT (BHQ1)-3′; IFN-γ, 5′-(FAM) CTTCTTCAGCAACAGCAAGGCGAA (BHQ1)-3′; IL-4, 5′-(FAM) TGGCGTCC CTTCTCCTGTGACCTCG (BHQ1)-3′; IL-10, 5′-(FAM) GCACCCACTTCCCAGTCGGCC AGAGCC (BHQ1)-3′; TGF-β,5′-(FAM) TTCAGCCACTGCCGTACfAACTCCAG (BHQ1)-3′; GAPDH, 5′-(FAM) CGTGCCGC CTGGAGAAACCTGCC (BHQ1)-3′.

2 µg total lung RNA of each animal from each treatment group at each time point was reverse transcribed in a volume of 20 µl using the following program:37°C for 15 min and 85°C for 5 s. 2 µl of cDNA was used in a 25 µl realtime PCR reaction volume. The difference of the amplification efficiency between the target gene and the housekeeping gene was identified by comparing the slopes of the standard curves. The PCR reactions were run on ABI 7500 (Applied Biosystems) using the following program: 95°C for 30 s, and 40 cycles of 95°C for 5 s and 60°C for 34 s. Analysis was performed using the 7500 system software (Applied Biosystems).

### Statistical analyses

The SPSS 16.0 software was used to conduct statistical analyses. The differences between values were evaluated through a one-way analysis of variance (ANOVA) followed by pair-wise comparison with the Student-Newman-Keuls test. P<0.05 was considered statistically significant and all values are means ±SEM.

## Supporting Information

Figure S1 **The localization of CD4+Foxp3+ T cells in lung tissue examined by immunofluorescence (x400).**
The CD4+Foxp3+ T cells were colocalized in the lung tissue sections by confocal immunofluorescence microscope, analyzed with the leica confocal software package. a1-a3, day 7; b1-b3, day 28 and c1-c3, day 56. a1-c1, saline group; a2-c2, silica group; a3-c3, silica+anti-CD25 mAb.
(TIF)

Figure S2 **Treg cells suppress the Th1 cytokine (IFN-γ) in the mice model of silica-induced lung fibrosis.**
IFN-γ cytokine was assayed by real-time RT-PCR by using -△△Ct method. (n = 5) (*, as compare with the saline control group, P<0.05; △, as compare with the silica group, P<0.05; #, as compare with 7day of the same group, P<0.05).
(TIF)
