# Supplementary figures and images for: CD4+CD25+Foxp3+ Regulatory T Cells Depletion May Attenuate the Development of Silica-Induced Lung Fibrosis in Mice
Source: PLoS One. 2010 Nov 3;5(11):e15404. doi: 10.1371/journal.pone.0015404 (PMC2972313; doi:10.1371/journal.pone.0015404)

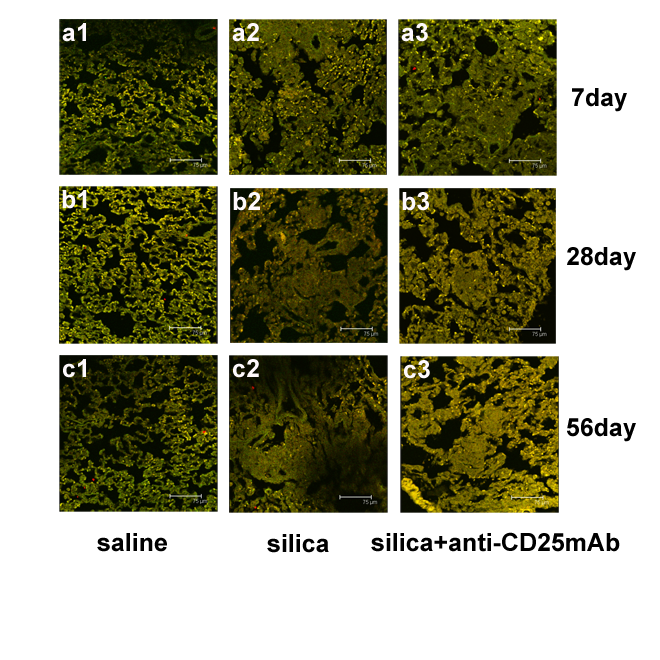

Supplement: Figure S1 — The localization of CD4+Foxp3+ T cells in lung tissue examined by immunofluorescence (x400). The CD4+Foxp3+ T cells were colocalized in the lung tissue sections by confocal immunofluorescence microscope, analyzed with the leica confocal software package. a1-a3, day 7; b1-b3, day 28 and c1-c3, day 56. a1-c1, saline group; a2-c2, silica group; a3-c3, silica+anti-CD25 mAb. (TIF) [file pone.0015404.s001.tif]

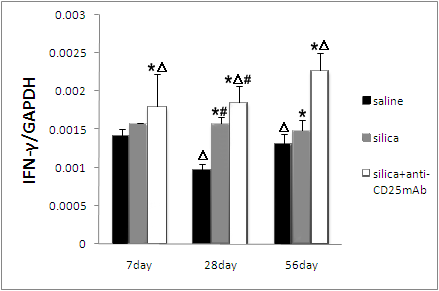

Supplement: Figure S2 — Treg cells suppress the Th1 cytokine (IFN-γ) in the mice model of silica-induced lung fibrosis. IFN-γ cytokine was assayed by real-time RT-PCR by using -△△Ct method. (n = 5) (*, as compare with the saline control group, P<0.05; △, as compare with the silica group, P<0.05; #, as compare with 7day of the same group, P<0.05). (TIF) [file pone.0015404.s002.tif]
